# *Cassia tora* Linn Cream Inhibits Ultraviolet-B-Induced Psoriasis in Rats

**DOI:** 10.5402/2012/346510

**Published:** 2012-03-25

**Authors:** Manmohan Singhal, Niraj Kansara

**Affiliations:** School of Pharmaceutical Sciences, Jaipur National University, Jaipur, Rajasthan 302025, India

## Abstract

The aim of present study was to determine the antipsoriatic activity of newly formulated O/W creams of methanolic extract of *Cassia tora* L. leaves by using ultraviolet-B-induced psoriasis in rat. The plant *Cassia tora* L. is traditionally claimed to be useful in the treatment of a number of skin diseases. However, there are no established scientific reports for its antipsoriatic activity. Methanolic *Cassia tora* L. leaves extract was used to prepare various concentrations of O/W creams and tested for acute dermal toxicity study. The different O/W creams showed good physical characteristics and passed the sensitivity, irritation, grittiness and bleeding test. The results of acute dermal toxicity showed that the creams were safe up to the dose of 2000 mg/kg. In case of psoriasis model, histopathological analysis revealed that there were absence of Munro's microabscess, elongation of rete ridges, and capillary loop dilation in the section in Test 2 (0.1%) and standard group. O/W creams and methanolic extract of *Cassia tora* L. leaves exhibited significant reduction in percentage of relative epidermal thickness and spleen index as compared to positive control. We concluded that topical O/W creams and crude extract containing methanolic extract of *Cassia tora* L. leaves have potent antipsoriatic activity in ultraviolet-B-induced psoriasis in rat.

## 1. Introduction

Psoriasis is a chronic, recurrent, inflammatory skin disease that affects 2-3% of the population worldwide and causes significant morbidity and mortality. Classic lesion is a well-marginated, redness of the skin due to pathological changes, erythematous plaque with silvery-white surface scale, mainly distributed into extensor surfaces (knees, elbows, buttocks) and may also involve palms and scalp. Associated findings also include psoriatic arthritic and nail changes. There are inflammation, hyperproliferation of the epidermis, and vascular alterations which lead to the redness. Its exact etiology is unknown, but it is generally believed to be a complex autoimmune inflammatory disease with a genetic basis [1]. Histologically, psoriasis is characterized by acanthosis (thickened epidermis) and parakeratosis (nucleated cells in stratum corneum) and has been described as showing benign hyperplasia. The dermal blood vessels are abnormally tortuous and dilated, and lymphocytic infiltration is frequently seen in the dermis and occasionally in the epidermis [2]. Therefore, some effective therapies appear to act as antiproliferative agents and diminished rates of either epidermal DNA synthesis, mitosis or both. Treatment of psoriasis includes topical, systemic, biological, and phototherapy [3]. But these therapies have many side effects. So, there is requirement of an alternative therapy like natural remedies.

Herbal extract is used to prepare cosmetic preparations to treat different skin disorders, augmenting beauty. Herbal extracts provide an idea to develop new herbal formulation for hyperpigmentation. Topical creams are used to enhance the solubility and bioavailability of therapeutic drugs. The plant *Cassia tora *L. (Caesalpiniaceae) is traditionally claimed to be useful in the treatment of Psoriasis and other skin diseases [4, 5]. *Cassia tora *L. leaves enriched in glycosides and also containing aloe-emodin may be beneficial for the skin diseases [6]. However, there are no established scientific reports for its antipsoriatic activity. Hence, the plant *Cassia tora *L. has been chosen to establish scientific data for its traditional claim as antipsoriatic. 

## 2. Materials and Methods

### 2.1. Chemicals

Standard (Tretinoin-0.05%) cream was obtained from Ethnor Pharma. Diethyl ether was obtained from Rankem, India. Other ingredients such as light liquid paraffin (Astron), cetostearyl alcohol (Chemdyes), propylene glycol (Nomex), white soft paraffin (Nomex), butyl hydroxyl toluene (Rankem), benzyl alcohol (Chemdyes), disodium EDTA (Rankem), isopropyl myristate (FD Fine Chemicals), and dibasic potassium phosphate (Rankem) were used to prepare O/W creams. For histopathological analysis, formalin (Merck), hematoxylin (Span Diagnostics), and eosin (Span Diagnostics) were used.

### 2.2. Plant Material

Leaves of *Cassia tora* L. (Cesalpinaceae) were collected from Dabhoi, Dist. Vadodara, Gujarat, India and authenticated by Profssor Dr. P. S. Nagar, Department of Botany, The Maharaja Sayajirao University of Baroda, Vadodara, Gujarat, India. The voucher specimen (03PG768, Niraj) has been deposited in the herbarium section of the Botany Department, The Mahararaja Sayaji Rao University of Baroda, Vadodara, Gujarat for future and further reference. The leaves were dried in shade and crushed in the grinder, coarse powder used for extraction.

### 2.3. Preparation of Extracts

The methanolic extract was prepared by cold maceration method [7] by taking 200 g of powdered leaves and extracting with 600 mL of methanol for 4 days. Extract was filtered; filtrate was evaporated, using a rotary evaporator under reduced pressure to dryness. The extract was used to prepare different concentration of O/W creams.

### 2.4. Formulation Development

Aqueous phase consisting of water (q.s) was heated to the temperature (70 ± 5°C) and then add disodium EDTA (0.01%), butyl hydroxyl toluene (0.001%), dibasic potassium phosphate (0.2%) in it. Then *Cassia tora *L. extract (0.05%, 0.1%, and 0.2%) was mixed in benzyl alcohol (1%) and added in it. After that, oily phase was added to the aqueous phase with continuous stirring at slow speed for 1 hour and slowly decrease temperature and mean while add isopropyl myristate (4%) in the mixtures of both phases. O/W creams were prepared by the addition of oily phase to the aqueous phase with continuous agitation. Oily phase consisted of light liquid paraffin (8%), cetostearyl alcohol (10%), propylene glycol (5%), glycerin (5%), white soft paraffin wax (12%), polyethylene glycol 4000 (5%), tween 80 (5.33%), and butyl hydroxyl anisole (0.001%). The prepared creams were transferred into wide mouth containers and stored in cool place. Base was also prepared by the same above method and with same ingredients but without *Cassia tora *L. extracts.

## 3. Evaluation of O/W Creams [8]

### 3.1. Physical Evaluation

1. Color
2. Odour
3. Form of physical state
4. pH: pH of the prepared formulation was measured using digital pH meter.
5. Net Content: Weigh the packed product then weigh without product. The difference of weight gives net content weight.

**Sensitivity Test**

It is tested by “Patch test.” Apply product on 1 cm^2^  patch of skin; if there is no any inflammation or rashes then it is considered as free from sensitivity.

**Irritation Test**

It is carried out by applying product on the skin for 10 minutes. If there is no irritation then it is considered as non-irritating product.

**Grittiness**

A pinch of product is rubbed on skin and then observed with magnifying glass; if it is free from rashes or eruption then it is considered as free from grittiness.

**Bleeding Test**

Such type of evaluation is carried out for semisolid preparation. The products are kept frequently for a period of time alternatively in fridge and at room temperature then bleeding of liquid is observed; if no liquid phase is omit out then it is considered as stable product for climatic conditions.

**Stability Studies**

The International Conference on Harmonization (ICH) harmonized tripartite guidelines on stability testing of new drug substance and product was issued on October 27, 1993. The formulated O/W creams and cream base were filled in the wide mouth containers and stored at 40°C ± 2°C/75% RH ± 5% RH for a period of three months.

**Animals**

Adult albino male mice (weight: approx. 25–27 g.) were used for the experiment. Animals were kept in the Shree Dhanvantary Pharmaceutical Analysis and Research Centre, Kim, Surat after approval from the Institutional Animal Ethical Committee (Reg. no.1103/abc/07/CPCSEA), were housed in polypropylene mouse cages as 3 animals will be housed per cage and rice husk will be used as the bedding material with a 12 h light-dark cycle, at temperature of 22 ± 02°C, humidity 30–70%, and kept laboratory mouse pellet feed (Pranav Agro Ltd.) and pure drinking water will be supplied *ad libitum. *The animals will be acclimatized to the laboratory conditions for a minimum period of seven days prior to commencement of treatment.

**Acute Dermal Toxicity Study**

The acute dermal toxicity test (LD50) of cream was determined according to the OECD guidelines no. 402 (Organization for Economic Corporation and Development) [9]. Adult Wistar rats (approx. 250–300 g.) of either sex were used. Approximately 24 hours before the test, fur should be removed from the dorsal area of the trunk of the test animals by clipping of shaving. Not less than 10% of the body surface area should be clear for the application of the test substance. Starting dose of 2000 mg/kg (topically) of cream was given to three groups (*n* = 6) each. Cream should be held in contact with the skin with a porous gauze dressing and non-irritating tape throughout a 24-hour exposure period. The treated animals were monitored for 14 days for changes in fur, eyes, behavior, and toxic reactions. The cream was safe up to the dose of 2000 mg/kg and from results suitable dose was chosen for each activity in each cream for further experimentation.

## 4. Evaluation of Antipsoriatic Activity in UV-B Induced Psoriasis in Rats [10]

### 4.1. Assessment of Different Formulations and Extract on UV-B-Induced Psoriatic Rats

 The animals were treated with respective doses of different concentrations of O/W creams (Test 1—0.05%, Test 2—0.1%, Test 3—0.2%), standard (Tretinoin-0.05%), cream base, and crude extract applied topically (single dose) during whole treatment. The animals were divided into seven groups as follows.

Group 1: Positive control.
Group 2: Standard (Tretinoin-0.05%) cream (topical).
Group 3: Test 1 (0.05%) cream (topical).
Group 4: Test 2 (0.1%) cream (topical).
Group 5: Test 3 (0.2%) cream (topical).
Group 6: Cream base (topical).
Group 7: Extract (topical).

Wistar rats (male, 300 g) were selected and divided into seven groups. Hair on the dorsal skin was carefully shaved. Test creams were applied topically on the dorsal part of the skin exposed to radiation. An area (1.5–2.5 cm) on one side of the flank is irradiated for 15 min (1.5 J/cm^2^) at a vertical distance of 20 cm with UV-B lamps. A biphasic erythema is observed. The second phase of erythema starts 6 h after the irradiation and gradually increases, peaking between 24 and 48 h. The color is brownish-red, and the reaction is confined to the exposed area with sharp boundary. By 48–72 h after irradiation, dark-brown scale is formed on the erythematous lesion. The irradiated rats are sacrificed after various time intervals by decapitation under ether anesthesia. Skin biopsies are taken immediately, fixed in 10% formalin, and embedded in paraffin. Tissue sections (4 *μ*m thick) are stained with hematoxylin and eosin.

### 4.2. Histopathological Examination

1. Sections were examined for presence of Munro's microabscesss, elongation of rete ridges, and capillary loop dilation by direct microscopy.
2. It was also examined the vertical epidermal thickness between the dermoepidermal junction and the lowest part of the stratum corneum (*n* = 3 measurements per scale, *n* = 3 scales per animal, *n* = 6) The percentage relative epidermal thickness of all the groups was calculated in comparison to the positive control group (100%; *n* = 54 measurements per treatment).

### 4.3. Immunological Analysis

**Spleen Index**

Spleen index was determined from the weight of the spleen of the mice surviving up to 10 days. On the 10th day of treatment, 6 mice from each of the seven groups were sacrificed and the spleens were recovered and weighed. The results are expressed as the organ index using the formula: weight of spleen (g)/body weight (g) × 100.

**Statistical Analysis**

All the experimental results were expressed as mean ± SEM. For statistical comparisons, explorative probabilities were obtained by the analysis of variance (ANOVA) followed by Dunnett's multiple comparison test using GraphPad Prism 5 (GraphPad software, Inc).

## 5. Results and Discussion

### 5.1. Evaluation of Cream

Three different concentrations of O/W creams (Test 1—0.05%, Test 2—0.1%, and Test 3—0.2%) were prepared to evaluate antipsoriatic activity. Numbers of parameters were performed to evaluate O/W creams. Physical evaluation revealed that creams having light green colour, characteristic odour, semisolid in nature, and pH ranged from 6.5 to 7. They passed the sensitivity test, irritation test, grittiness, and bleeding test. Stability of creams (base and formulation) was evaluated on 40°C ± 2°C/75%, RH ± 5% RH for a period of three months. No phase separation was observed during the stability study of creams. No liquefaction is observed throughout the study period of three months.

### 5.2. Acute Dermal Toxicity Study

The acute dermal toxicity test (LD50) of creams was determined according to the OECD guidelines no. 402 (Organization for Economic Corporation and Development). The creams were safe up to the dose of 2000 mg/kg. There were no changes in fur, eyes, and behavior of treated animals as well as no toxic reactions determined and from results suitable dose (250 mg (0.05%), 500 mg (0.1%), and 1000 mg (0.2%)) was chosen for each activity in each cream for further in vivo studies.

### 5.3. Evaluation of Antipsoriatic Activity

Screening of antipsoriatic activity was carried out by topical application of different concentration of O/W creams, cream base, methanolic extract of *Cassia tora *L. leaves, and Standard (Retino-A (Tretinoin cream-0.05%)) by using ultraviolet-B-induced psoriasis in rat.

### 5.4. Histopathological Examination

**Figure 1:**
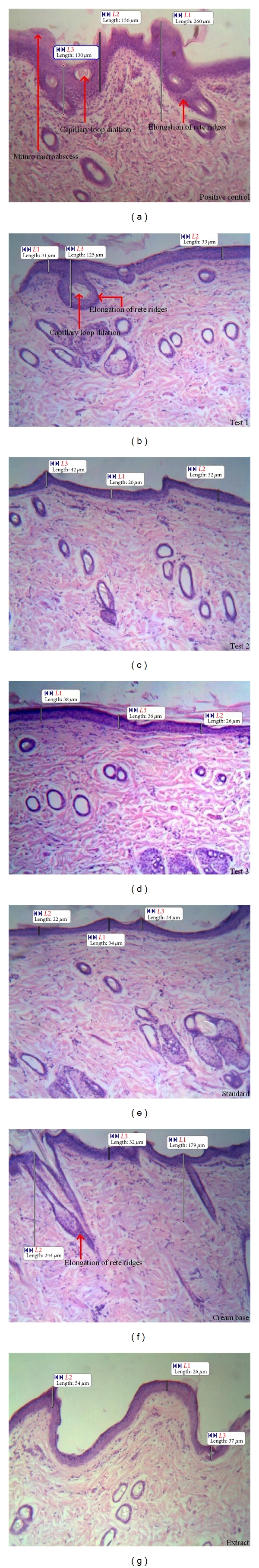
Effect of different formulations on histopathological features on U.V.-B-induced psoriasis in rats.

Histopathologically, numbers of features are observed in fully developed lesions in psoriasis such as Munro's microabscess, regular elongation of rete ridges, and capillary loop dilation which are shown in Table 1 and Figure 1. 

In case of positive control group, section showed regular elongation of rete ridges, capillary loop dilation with minimal grade lesion of diagnostic Munro's microabscess and marked increase in relative epidermal thickness as compared to other groups. In case of Standard group, there was absence of Munro's microabscess, capillary loop dilation along with elongation of rete ridges in the section showing good therapeutic effects. In case of Test 1 group, there was slight decrease in elongation of rete ridges, minimal grade lesion of Munro's microabscess along with capillary loop dilation in the section. In Test 2 group, there was no lesion of Munro's microabscess, capillary loop dilation along with elongation of rete ridges in the section of skin of rats. In case of Test 3 group, there was decrease in elongation of rete ridges and absence of Munro's microabsces as well as capillary loop dilation. Cream-base-treated group showed mild grade lesions of elongation of rete ridges and minimal grade lesion of Munro's microabscess as well as capillary loop dilation. Extract treated group showed absence of Munro's microabscess and capillary loop dilation and minimal grade lesion of elongation of rete ridges. 

### 5.5. Relative Epidermal Thickness

**Figure 2:**
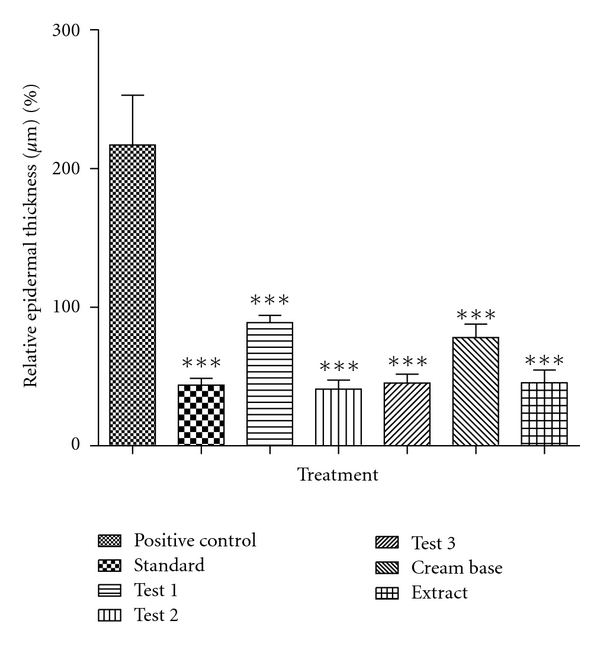
Effect of different formulations on relative epidermal thickness on U.V.-B induced psoriasis in rats.

In comparison to Positive control group, all other groups led to significantly decreased relative epidermal thickness. Statistical analysis for the relative epidermal thickness revealed the following range of efficacies in the induction of epidermal differentiation: Test 2 > Standard > Test 3 > Extract > Cream base > Test 1 > Positive Control. The results of epidermal thickness are presented in the Table 2 and Figure 2.

### 5.6. Immunological Analysis

#### 5.6.1. Spleen Index

**Figure 3:**
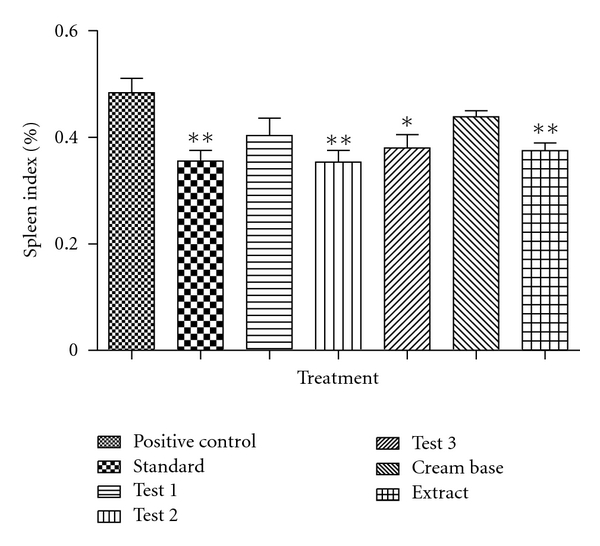
Effect of different formulations on spleen index on U.V.-B-induced psoriasis in rats.

In comparison to positive control group, all other groups led to significantly decreased spleen index. The effect of different formulations on spleen index is shown in Table 3 and Figure 3.

## 6. Conclusion

From the preliminary study we concluded that stable topical O/W creams containing methanolic extract of *Cassia tora *L. leaves significantly and dose dependently decreased in the relative epidermal thickness of animal skin as well as other histopathological features and also suppressed spleen index in *in vivo *studies. Topical O/W creams and crude extract containing methanolic extract of *Cassia tora *L. leaves have potent antipsoriatic activity. The present investigation aims at the development of potent phytomedicine for treatment of psoriasis from the *Cassia tora *L. plant.

## Figures and Tables

**Table 1:** Effect of different formulations on Histopathological features on U.V.-B-induced psoriasis in rats.

Treatment	Munro's microabscess	Elongation of rete ridges	Capillary loop dilation
Positive control	+	+++	++
Standard	−	−	−
Test 1	−	+	+
Test 2	−	−	−
Test 3	−	+	−
Cream base	+	++	+
Extract	−	+	−

*Notes.* **+ **mild or slight grade lesion; **++ **moderate grade lesion; **+++ **severe grade lesion; − no lesion in.

**Table 2:** Effect of different formulations on Relative Epidermal thickness on U.V.-B-induced psoriasis in rats.

Sr. no.	Treatment (topical)	% relative epidermal thickness (*μ*m) Mean ± SEM
(1)	Positive control	100 ± 35.77
(2)	Standard	20.17 ± 4.99***
(3)	Test 1	40.92 ± 5.39***
(4)	Test 2	18.82 ± 6.69***
(5)	Test 3	20.93 ± 6.39***
(6)	Cream base	35.99 ± 9.57***
(7)	Extract	20.94 ± 9.26***

*Notes.* Each value represents Mean ± SEM, *n* = 6, ***P < 0.001 compared to Positive control group. One-way ANOVA followed by Dunnett's test.

**Table 3:** Effect of different formulations on spleen index on U.V.-B-induced psoriasis in rats.

Sr. no.	Treatment (topical)	% spleen index Mean ± SEM
(1)	Positive control	0.4833 ± 0.03
(2)	Standard	0.3550 ± 0.02**
(3)	Test 1	0.4033 ± 0.03
(4)	Test 2	0.3533 ± 0.02**
(5)	Test 3	0.3880 ± 0.02*
(6)	Cream base	0.4383 ± 0.01
(7)	Extract	0.3750 ± 0.01**

*Notes*. Each value represents Mean ± SEM, *n* = 6, *P < 0.05; **P < 0.01 compared to Positive control group. One-way ANOVA followed by Dunnett's test.
